# Furazolidone susceptibility of Helicobacter pylori isolated from patients with gastroduodenal diseases in Colombia

**DOI:** 10.1590/S1678-9946202567020

**Published:** 2025-04-04

**Authors:** Brandonn Lopera, Kellys Lemos, José Danilo Atehortúa, Joaquín Valencia-Cárdenas, Diego Vélez-Gómez, Alonso Martinez, Tania Pérez-Cala, Beatriz Salazar

**Affiliations:** 1 Universidad de Antioquia, Escuela de Microbiología, Medellín, Colombia; 2 Universidad de Antioquia, Facultad de Medicina, Departamento de Microbiología y Parasitología, Grupo Bacterias & Cáncer, Medellín, Colombia; 3 Universidad de Antioquia, Hospital Alma Mater, Medellín, Colombia

**Keywords:** Helicobacter pylori, Furazolidone, Drug resistance, Microbial, Stomach diseases, Colombia

## Abstract

Estimates suggest that over 50% of the global population suffer from *Helicobacter pylori* infections. Nowadays, first-line quadruple therapy is recommended to eradicate the bacteria due to the increasing failures of the standard triple therapy. Thus, antibiotics such as furazolidone have emerged as a new treatment due to their success rate (>90%) in rescue therapies. Nevertheless, furazolidone is not routinely used for treatment of *H. pylori* in Colombia. Still, some Asian countries commonly prescribe it. This study aimed to determine the susceptibility of *H. pylori* to furazolidone in isolates from patients with gastroduodenal diseases in Colombia that were extracted from 2019-2022. A descriptive study was carried out with 179 patients with gastroduodenal diseases. Susceptibility was determined by the agar dilution method. The gene *oorD* from resistant isolates was amplified by polymerase chain reaction, and their PCR products were sequenced. The frequency of *H. pylori* equaled 23.5% (42/179); the bacterium was isolated in 84 gastric biopsies. Moreover, 1.7% (3/179) of patients had one resistant isolate to furazolidone in at least one of the two gastric biopsies, corresponding to 5.95% (5/84) of the isolates resisting furazolidone. Overall, three new mutations in the *oorD* gene occurred in one isolate, and two of the mutations in the five isolates had been reported in Iran, Brazil, and China. This research found low *in vitro* resistance of *H. pylori* isolates to furazolidone in Colombia. Finally, all five isolates showed mutations in the *oorD* gene.

## INTRODUCTION

*Helicobacter pylori (H. pylori)* constitutes the second most prevalent bacterium in the world. Estimates suggests that more than 50% of the world population is infected with it. Epidemiological studies have shown that the infection is common in low- and middle-income countries. It is also associated with factors such as socioeconomic level, overcrowding, age, sex, among others^1^. On the other hand, 15-20% of those infected develop diseases such as chronic atrophic gastritis, peptic ulcer, mucosa-associated lymphoid tissue lymphoma, and gastric cancer (GC). In 1994, the World Health Organization classified the bacterium as a grade I carcinogen since 90% of patients with GC have been previously infected with *H. pylori* ^2^.

Several therapeutic schemes aim to eradicate *H. pylori*. Currently, the most recommended treatment refers to quadruple therapy due to the limitations of the standard triple therapy, which combines a proton pump inhibitor with two antibiotics, such as amoxicillin, metronidazole, and/or clarithromycin^3^. Studies carried out in various regions of Colombia — including Bogota, Pereira, Armenia, Manizales, Tumaco, and Antioquia — have shown therapeutic failure rates of 20-30% with the triple therapy regimen^4^. This therapy fails to reach a 90% efficacy due to factors such as underdosing, lack of adherence to treatment, use of empirical treatments, previous consumption of antibiotics, among others^5,6^. The latter makes the therapeutic approach difficult, generating resistance to antimicrobials; hence the need to seek therapeutic alternatives such us furazolidone^3^.

Furazolidone serves as salvage therapy after the failure of three previous eradication therapies. This antibiotic (a nitrofuran) has been used for more than 30 years as a treatment against bacterial and protozoal infections, showing safety and a broad spectrum^3,8^. A 2019 Chinese study with 584 participants found that furazolidone therapies showed 91.8% efficacy^8^. In Colombia, the use of furazolidone in rescue or second-line therapies remains uncommon^7^.

Resistance to furazolidone varies across the world. However, it is mostly low (<15%)^9^. Nevertheless, there exist insufficient data on its efficacy and resistance in Colombia^6^, South American^10^, the Caribbean^11^, and the United States^9^. South American studies have found an 86-100% susceptibility of *H. pylori* to furazolidone^10,12-14^, making it an alternative to the high antibiotic resistance of *H. pylori* to clarithromycin and metronidazole (according to the Maastricht VI Consensus)^15^ — especially in Colombia (which shows worrisome rates of resistance to clarithromycin, metronidazole, and amoxicillin^4^) —, especially due to its low cost^14^. To date, China and Iran have carried out the largest number of studies on the resistance of *H. pylori* to furazolidone^9^. A 2022 Chinese study with 2,772 isolates of *H. pylori* determined that 99.7% showed sensitivity to furazolidone^16^; whereas other research in Iran from 2022 with 20 isolates an 85% sensitivity^17^. In Colombia, only one study has evaluated susceptibility to furazolidone, which was carried out in Narino, Colombia in 2024. It evaluated 239 *H. pylori* isolates and showed a 99.2% genotypic sensitivity to furazolidone^18^.

On the other hand, only two studies have assessed genotypic and phenotypic resistance to furazolidone, hence the limited knowledge about mutations associated with resistance to this antibiotic. Concerning the molecular mechanisms of resistance to furazolidone in *H. pylori*, some genetic mutations have been identified in the 2-oxoglutarate acceptor oxidoreductase (*oorD*) gene, including A041G, A122G, C349A(G), A78G, A112G, A335G, C156T, and C165T^11,19,20^.

Considering the above, it is essential to determine the susceptibility of *H. pylori* to furazolidone to provide useful information that can implement effective therapeutic measures following microbiology laboratory results that minimize the development of resistance. Therefore, this study aimed to determine the susceptibility to furazolidone in *H. pylori* isolates from three regions in Antioquia (Colombia), which were extracted from 2019 to 2022.

## MATERIALS AND METHODS

### Type of study and sample size

A descriptive, cross-sectional study was carried out. To calculate the sample size, a universe of 515 individuals who were treated in one year (42 patients/month) was considered based on data provided by the participating health entities. The estimated frequency of *H. pylori* infection equaled 64%, based on previous studies in the region^1,21^. A confidence interval of 95% and a sampling error of 5.7% were considered. A sample of 179 participants was estimated using the software Epi Info, version 7.

### Patient recruitment and selection criteria

**Figure 1:**
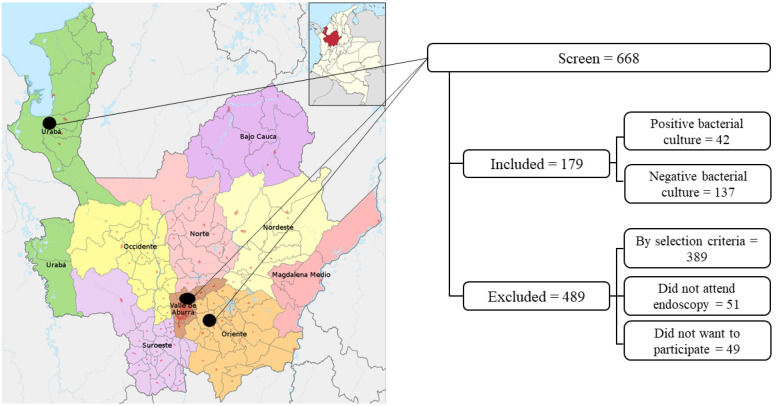
Patients with gastroduodenal diseases included in this study.

The study population consisted of individuals aged over 18 years who sought the upper gastrointestinal endoscopy services of eight health entities in three regions of Antioquia, Colombia (Metropolitan Area of the Aburra Valley, the Easter subregion, and Uraba) (Figure 1). Participants may have shown the symptomatology of gastroduodenal disease. Patients with GC were considered given the importance of the association between infection and the development of the disease, as were participants with or without prior treatment for *H. pylori* infection.

Pregnant women, individuals with coagulation disorders or active hemorrhage, and patients with serious concomitant diseases such as tumors or other malignant diseases (except GC) were excluded from this study. Other excluded individuals referred to people who received proton pump inhibitors and/or H2 receptor antagonists in the last 15 days prior to endoscopy, antibiotics for *H. pylori* during the last month, radiotherapy, chemotherapy, and patients with mental alterations that prevented participation in this study.

### Ethics approval and consent to participate

This study was approved by the Institutional Review Board of the Faculty of Medicine of the University of Antioquia in Act 016 of 2019, Fundacion Hospitalaria San Vicente de Paul in Acta 28-2021, and IPS Universitaria in Acta CTI IN44-2019. Participants signed the informed consent that was approved by these committees. This research was performed in accordance with the Declaration of Helsinki guidelines. All the materials and reagent sources in this study are described in the methods section.

### Data collection and analysis

Participants signed an informed consent form. An electronic survey was conducted in the Kobocollect^®^ application, with questions about participants’ sociodemographic aspects, lifestyles, hygienic conditions, and personal and family clinical history. The obtained data and the results of the susceptibility tests were exported onto SPSS^®^ (version 25, IBM, USA), in which descriptive statistics were carried out with measures of central tendency, frequency, and summary tables.

### Sample collection

To collect the gastric biopsies, the endoscopic surgeons extracted two samples: one from the antrum and another from the corpus of each patient. Samples were taken with an OLYMPUS^®^ endoscope with reusable gastric biopsy forceps (sterile for each patient). Once extracted, they were deposited into BD BBL™ Brucella broth supplemented with 10% (v/v) fetal bovine serum (Gibco, USA) and 10% (v/v) glycerol (Sigma Aldrich, Malaysia). The tubes were stored in a freezer with dry ice at −20 °C for transportation. The samples were evaluated in the microbiology laboratory for culture and susceptibility tests.

### Culture and identification

The biopsies were deposited in tubes with 250 μL of sterile 0.9% Corpaul^®^ saline solution that were homogenized with a Fisherbrand^®^ electric mortar and confluently seeded on Brucella^®^ agar supplemented (Becton Dickinson, USA) with BD BBL™ IsoVitalex^®^ 0.4% (v/v) (Becton-Dickinson, USA) and DENT^®^ Oxoid 0.2% (v/v). The seedlings were incubated under microaerophilic conditions (5-10% O_2_, 5-10% CO_2_, 80-90% N_2_, and 95% humidity) for 96 h until compatible colonies could be seen. Then, identification tests were carried out to confirm the presence of *H. pylori* (Gram staining, catalase, urease, and oxidase)^22^.

### Agar dilution method

#### Antibiotic preparation

Sigma-Aldrich^®^ furazolidone (Sigma-Aldrich, St. Louis, MO, USA) was used following the general recommendations from the Clinical & Laboratory Standards Institute (CLSI) guideline M100 E32, 2022^23^. The weight of the antibiotic was calculated without reconstitution to obtain a stock solution of 5,120 μg/mL in dimethyl sulfoxide-Sigma-Aldrich^®^, which was bottled in 1 mL cryovials and stored at −70 °C. From this solution, serial 1:2 dilutions were made from a concentration of 40 μg/mL to a concentration of 0.3125 μg/mL as follows: 40; 20; 10; 5; 2.5; 1.25; 0.625; and 0.3125 µg/mL) using 1X PBS as a diluent (pH 7.4).

#### Culture medium preparation

Each concentration of the antibiotic was deposited on BD BBL™ Müeller-Hinton II (AMH-II) agar supplemented with 10% (v/v) horse blood and 0.4% (v/v) of BD BBL™ IsoVitalex^®^ in a 1:10 dilution of the culture in relation to the antibiotic obtaining a final concentration of 4.0 to 0.03125 μg/mL of the antibiotic^23,24^. Finally, each antibiotic concentration was deposited in 90 × 15 mm Petri dishes.

#### Bacterial inoculum preparation

A 2 McFarland Standard was prepared (≈ 1×107 to 1×108 CFU/mL) in 0.9% saline solution of each bacterial isolate, which was subcultured twice with growth for 72-96 h on supplemented Brucella BD BBL™ agar with 7% (v/v) horse blood and 0.4% (v/v) BD BBL™ IsoVitalex^®^.

#### Inoculum preparation

The procedures were carried out according to the recommendations of the CLSI guidelines^24^. To optimize the inoculum, different volumes were evaluated: 1, 2, 5, 10, and 20 μL using an automatic pipette that replaced a steer replicator. In each antibiotic concentration, the volume of the inoculum was deposited vertically on the agar, leaving a seeding spot for each isolate (up to 12 different isolates were seeded in each 90 × 15 mm Petri dish).

The different concentrations of the antibiotic (0.03125-4 μg/mL) were evaluated in clinical isolates. The ATCC 43504 (American Type Culture Collection) and NCTC 11637 (National Collection of Type Cultures) strains of *H. pylori* were used as a control. The use of the ATCC 43504 strain is recommended as a control by the CLSI M100 and CLSI M45 guidelines for susceptibility assays^23,24^. The NCTC 11637 strain was chosen following the same premise, and its nomenclature is given by the European Agency UK Health Security. For the negative control, the same strains were evaluated. However, instead of incubating under optimal growth conditions (72-96 h at 37 °C in microaerophilic conditions), they were brought to temperatures from 2 to 4 °C for four to five days^23,24^. The Minimum Inhibitory Concentration (MIC) was considered the lowest dilution that completely inhibited bacterial growth when compared to the positive control. The cut-off point equaled 0.5 μg/mL^25,26^. Values above this were defined as resistant isolates^25^.

#### PCR and sequencing

To amplify the *oorD* gene, the primers F: 5’- TTTAGCACAAAGGAGAATG −3’ and R: 5’- AACTTGGCGTAATAGGAT −3’ were used as per Su *et al*.^19^.PCR reactions were carried out in a final volume of 20 µL with the Taq polymerase Master Mix kit (New England Biolabs. Inc, Hitchin, Herts, UK). The PCR was amplified in the thermocycler (Labnet International, Inc NJ, USA) under the following conditions: initial denaturation at 94 °C for 5 min, followed by 25 cycles. Each cycle was comprised of denaturation at 94 °C for 30 s, annealing at 50 °C for 30 s, and extension at 72 °C for 30 s, followed by a final extension at 72°C for 5 minutes. DNA from strains ATCC 43504 and NCTC 11637 were used as positive control; H_2_O was used as negative control. The amplicons were run on 3% agarose gels at 50 volts for 120 minutes and stained with HydraGreen™ (ACTgene–USA). Finally, they were visualized in a transilluminator (Molecular Imager^®^ Gel Doc™ XR System. BioRad Laboratories, USA).

The amplified fragments of the *oorD* gene were sequenced by the Sanger method in the GENES SAS laboratory. The products were purified and sequenced using the BigDye™ Terminator v3.1 Cycle Sequencing kit (Applied Biosystems, Massachusetts). To determine the variants, the obtained sequences were compared with those from the *oorD* gene reference strain for *Helicobacter pylori* (GenBank AF021094.1). The sequences were evaluated with SecScape (Applied Biosystems^®^ version 3), whenever the quality value of which exceeded 20. Subsequently, the variants in the sequences were related using the DNA Dynamo v1.615 (Bluetractor software).

## RESULTS

### Standardization of the agar dilution method

The evaluation of several volumes of inoculum showed that using a 2 McFarland standard in a volume of 10 μL obtained the inoculum required for the agar dilution. A quantitative colony count in independent experiments evaluated the concentration of the final inoculum, ensuring that lied within the range in the CLSI guideline^24^.

### Microbial culture and susceptibility profile for furazolidone

**Figure 2:**
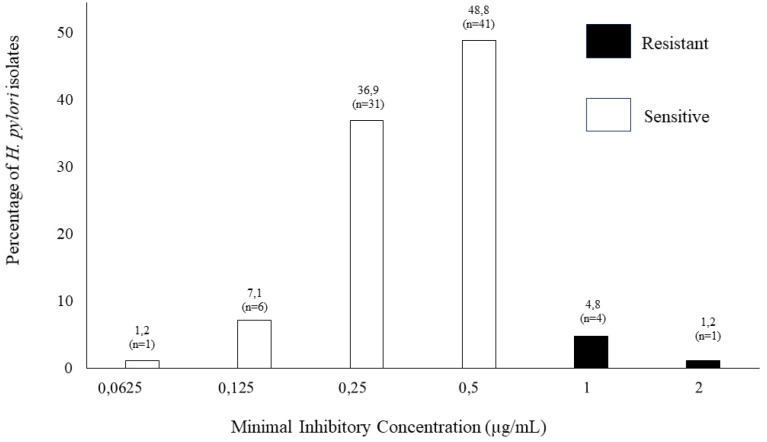
Distribution of furazolidone MIC in isolates from patients infected with *H. pylori*.

This study isolated *H. pylori* in 23.5% (42/179) of participants (95% CI: 17.8% - 30.2%) (Table 1), retrieving 84 isolates (42 from the antrum and 42 from the corpus). Of these isolates, 5.95% (5/84) resisted furazolidone (95% CI: 2.6% - 13.2%) (Figure 2). This corresponds to 1.7% (3/179) of patients having one resistant isolate to furazolidone in at least one of their two gastric biopsies. The range of susceptibility for both isolates (antrum and corpus from the same patient) coincided in all susceptibility cases.

**Table 1:** Sociodemographic description and personal history of gastroduodenal diseases of the population with *H. pylori.*

Variable	Categories	n (%) (N=42)
Sex	Men	11 (26.2)
	Women	31 (73.8)
Age	18-52	25 (59.5)
	53-87	17 (40.4)
Household income	< 1 MW	7 (16.6)
	≥ 1 y < 3 MW	27 (64.2)
	≥ 3 y < 6 MW	6 (14.2)
	≥ 9 MW	2 (4.8)
Occupation	Employed	13 (31.0)
	Housewife	18 (42.9)
	Independent	4 (9.5)
	Retired	3 (7.1)
	Student	2 (4.8)
	Unemployed	2 (4.8)
Socioeconomic status	Low (1 - 2)	16 (38.0)
	Medium (3 - 4)	23 (54.8)
	High (5 - 6)	3 (7.1)
Access to health services	Public	31 (73.8)
	Private	8 (19.0)
	Does not know	3 (7.1)
Location of the house	Urban	33 (78.6)
	Rural	9 (21.4)
Utility access	Gas	34 (81.0)
	Water supply system	41 (91.6)
	Sewage system	37 (88.1)
	Electricity	42 (100)
	Garbage collection	41 (91.6)
Signs and symptoms	Epigastralgia	36 (85.7)
	Nauseas	14 (33.3)
	Vomiting	5 (11.9)
	Dysphagia	12 (28.6)
	Globus sensation	14 (33.3)
	Dyspepsia	25 (59.5)
	Belching	73.8
	Reflux	25 (59.5)
	Loss of appetite	12 (28.6)
	Weight loss	8 (19)
	Hematemesis	0 (0)
	Melena	9 (21.4)
	Abdominal Distension	37 (88.1)
Diagnosis of gastric disease	Yes	34 (81.0)
	No	8 (19.0)
Previous endoscopy	Yes	28 (66.7)
	No	14 (33.3)
Previous diagnosis of *H. pylori* infection	Yes	26 (61.9)
	No	16 (38.1)

MW = National Minimum Wage per Month (For 2022, one MW is equivalent to USD 285/month). The table only shows data from positive samples (84/179) 23.5%.

### Amplicons and sequence analysis

**Figure 3:**
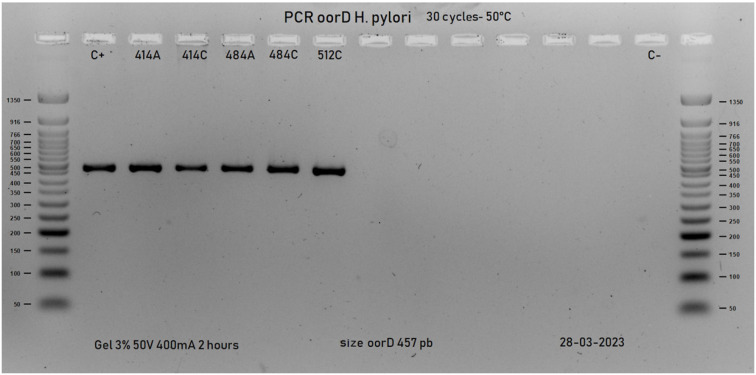
Agarose gel electrophoresis *oorD* gene.

**Figure 4:**
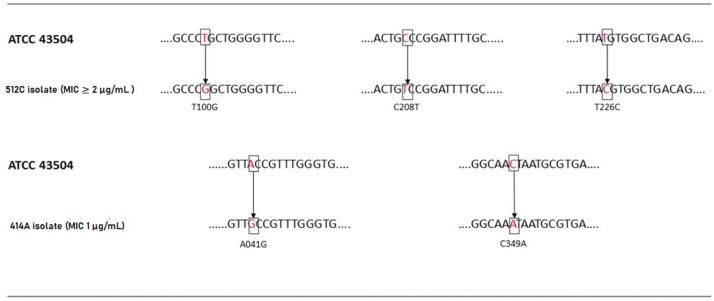
Analysis of *oorD* mutations from furazolidone-resistant strains of *H. pylori.*

This study obtained amplicons of the 457 bp *oorD* gene (Figure 3) in six isolates (five of which with phenotypic resistance to furazolidone and one ATCC 43504 strain). This research found five amino acid changes in the isolates with substitutions in A041G (Thr -> Ala), T100G (Leu -> Arg), C208T (Ala -> Val), T226C (Met -> Thr), and C349A (Thr-> Asn). It also found A041G and C349A in the five isolates with phenotypic resistance and T100G, C208T, and T226C in a single isolate [which had the highest MIC (2 µg/mL)] (Figure 4).

### Sociodemographic and personal history of gastroduodenal diseases

Out of the 179 participating individuals, 30.2% were men and 69.8% were women. Their average age equaled 59.6 years (from 19 to 87 years). All participants infected with *H. pylori* lived in a home with electricity (42/42). Concerning socioeconomic characteristics, 60.3% of participants belonged to socioeconomic status 1-3, and 80.9% of the infected population had a family income below three minimum wages.

### Characteristics of patients with isolates resistant to furazolidone

Of the patients, 1.7% (3/179) showed a furazolidone-resistant isolate. Regarding their characteristics, three participants lived on less than three minimum wages ($ 855 USD) and had access to running water from the municipal water supply system. Of them, two were women and one was a man, all of whom lived in an urban area. None smoked, and all showed gastric symptoms (3/3 epigastric pain and belching, 2/3 nausea and gastroesophageal reflux, and 1/3 emesis and anorexia) and had received a diagnosis of gastritis, whereas 2/3 had had previous infection with *H. pylori* and undergone antibiotic treatment.

## DISCUSSION

The agar dilution method is the gold standard the CLSI recommends to evaluate the antimicrobial susceptibility of *H. pylori* ^23,24^. Its advantage stems from its dispensing of special equipment and the ease of result interpretation^24^. However, the CLSI offers no cutting point to evaluate susceptibility to furazolidone in *H. pylori*. Therefore, this study interpreted its results based on previous research data^25,26^.

The *H. pylori* isolates obtained a 0.5-μg/mL cut-off point and a 94.0% sensitivity (79/84). Moreover, the results for the reference strains were reproducible. Similar results occurred by the same cutting point and method in others studies across the world^17,25,26^. In the Republic of Korea, Kwon *et al*.^25^ evaluated 431 *H. pylori* isolates and reported a susceptibility of 98.4%; Kohanteb *et al*.^26^ evaluated 106 isolates and reported a susceptibility of 90.6%. Currently, in Colombia, no other published study has reported phenotypic resistance to furazolidone; only one has evaluated genotypic resistance^18^, making it impossible to compare the cut-off point in Colombia. This would be the first study in the country to address phenotypic resistance.

Using the cut-off point of > 2 μg/mL, authors such as Mendonça *et al*.^12^, Su *et al*.^19^, Godoy *et al*.^27^, Kim *et al*.^28^, Sun *et al*.^29^, among others, found that the susceptibility of *H. pylori* to furazolidone ranged from 87.0 to 98.6%. This parameter was evaluated from 2000 to 2021 by the agar dilution method. This cut-off point is relevant for South America given that Brazil is the only country in the region that has evaluated resistance to this antibiotic in *H. pylori* ^10^, and most studies in Brazil use this cut-off point^12-14,27^.

The cut-off point in other studies equaled ≥ 4 μg/mL, including Alarcón *et al*.^30^, with a susceptibility of 98.2% in 164 isolates. Miftahussurur *et al*.^31^ found a sensitivity of 100% in 105 isolates, obtaining the same results in another study from the Dominican Republic using 64 isolates^11^, whereas Eisig *et al*.^32^ measured resistance to furazolidone in 39 isolates in Brazil, finding 100% of susceptibility. These are the only two publications in Latin America with this cut-off point.

## RESULTS

These results showed the evaluation of susceptibility patterns must have standardized reference ranges. However, the absence of routine evaluations offers a difficulty to antibiograms for furazolidone in *H. pylori*. Furthermore, cut-off points varied between studies: > 0.5 μg/mL^17,25,26^, ≥ 2 μg/mL^12,18,27-29^, and ≥ 4 μg/mL^11,30-31^. This study chose > 0.5 μg/mL as its cut-off point for one reason: Kwon *et al*.^25^ established it based on an evaluation of reference strains and clinical isolates as per the CLSI guideline^23^. Standardizing the method and establishing guidelines to evaluate the susceptibility of the microorganism to antibiotics are essential because they enable the reproducibility of the method, facilitate the comparison of studies, and establish a reference cut-off point^33^. Finally, establishing a universal cut-off point for this antibiotic in *H. pylori* is of paramount significance.

This study found different alterations in the *oorD* gene in the five furazolidone-resistant isolates of *H. pylori*, observing five nucleotide substitutions: A041G, T100G, C208T, T226C, and C349A; which had changed an amino acid in all of them. Su *et al*.^19^ had reported two of these mutations: A041G and C349A. Although these substitutions occur in all resistant isolates, their importance in furazolidone resistance remains unclear. Additionally, this study found, in only one isolate, three nucleotide substitution mutations at positions T100G, C208T, and T226C. These alterations were yet to be reported in Colombia^18^ or the world^19^. However, the highest MIC occurred in this isolate. For this reason, further studies are necessary to aid in document mutations linked to phenotypic resistance to furazolidone, particularly in South America. These studies will help establish the mechanisms underlying susceptibility loss and improve genotypic methods for detecting this resistance.

In the world, the prevalence of *H. pylori* infection is estimated to exceed 50%^1^. In Colombia, Bravo *et al*.^34^ found a frequency of 69.1%, with significant differences between regions, showing a high frequency in areas in the Andes Mountain range. In the department of Antioquia, the same study estimated a frequency at 65%, resembling the 64% in Salazar *et al*.^21^, which evaluated 272 individuals. In more recent studies in 2023, Salazar *et al*.^1^ found an infection frequency of 55.9%. The sociodemographic results in this study showed that all its participants had access to running water, energy, garbage collection, and sewage. Out of its population, 56.4% had a family income below three minimum wages, which coincides with previous studies in the region^21^. This research isolated *H. pylori* in 23.5% (42/179) of its participants, finding resistance to furazolidone in 5.95% (5/84) of isolates. Finally, two of the three patients with furazolidone-resistant isolates had a history of prior infection with *H. pylori* and had received treatment against this bacterium, which has been reported to reduce the rates of infection eradication^35^.

This study determined an infection frequency of 23.5% (42/179) by microbiology, which was lower than expected. This could be attributed to the COVID-19 pandemic, which made it difficult to recruit patients as gastric endoscopies and non-urgent surgeries decreased due to this emergency. Moreover, this research recruit most of its evaluated population from an institution that cares for patients with high incomes, in which infections occur less frequently^36^.

Although the frequency of infection in Colombia is high, 69.1% (from 55.9 to 88.7%)^1,21,34,37^, bacterial eradication therapies decrease their response to triple therapy due to the high resistance of *H. pylori* to the used antibiotics^4^. This can improve with the use of routine antibiograms or sequencing, which evaluates new antibiotics such as furazolidone and/or rifabutin^18,38^. It is essential to monitor susceptibility over time in all the isolates and evaluate the efficacy of alternative treatments that contribute to improving eradication therapies, especially in patients with recurrent therapeutic failure^3,38,39^. Finally, considering the cost-benefit ratio in relation to conventional triple therapy and its use in other countries in the region^14^, furazolidone would constitute a viable option in Latin America and in low-income countries as this antimicrobial would broaden the range of treatments against these bacteria.

Continuous evaluation of the susceptibility of this microorganism to different antibiotics is necessary given that *H. pylori* configures a bacterium of public health importance due to its association with gastroduodenal diseases and its growing resistance to antibiotics^2^. Improving eradication therapies becomes a priority for health workers. Thus, this important study provides data about high susceptibility to furazolidone and it generates important bases for future studies with this antibiotic. It may even serve to evaluate new antibiotics such as rifampicin by the same technique^39^. This study will hopefully contribute to the diagnosis and selection of treatment schemes that minimize the prevalence of infection and resistance rates of *H. pylori*.

## CONCLUSION

This is the third study in Colombia (and the first in Antioquia) to determine the susceptibility of *H. pylori* to this antibiotic. Of the 84 *H. pylori* isolates, 94.05% showed susceptibility to furazolidone, agreeing with previous studies. This study could assist the planning and development of protocols to determine the antimicrobial susceptibility of *H. pylori* using the agar dilution method recommended by CLSI. Finally, this study may aid future research in evaluating the susceptibility of the bacteria to furazolidone or other alternative antibiotics serving as unconventional treatments in patients with recurrent therapeutic failures.
